# Epidemiology of Meal‐Related Abdominal Discomfort or Pain in Irritable Bowel Syndrome

**DOI:** 10.1111/nmo.70174

**Published:** 2025-09-30

**Authors:** Melanie S. Cuffe, Vivek C. Goodoory, Cho Ee Ng, Christopher J. Black, Alexander C. Ford

**Affiliations:** ^1^ Leeds Gastroenterology Institute St. James's University Hospital Leeds UK; ^2^ Leeds Institute of Medical Research at St. James's University of Leeds Leeds UK; ^3^ County Durham and Darlington NHS Foundation Trust Durham UK

**Keywords:** diet, gut‐brain interaction, irritable bowel syndrome, quality of life, symptoms

## Abstract

**Background:**

Patients with IBS often report meal‐related symptoms, which may negatively affect IBS‐related quality of life, psychological health, and lead to food‐avoidant behaviors. However, the understanding of the epidemiology of these symptoms is limited.

**Methods:**

We compared characteristics of adult patients with Rome IV‐defined IBS with and without meal‐related abdominal discomfort or pain ≥ 50% of the time. Participants were recruited from the ContactME‐IBS research register. We collected data concerning demographics, IBS symptoms, psychological health, quality of life, and impact on work and daily activities using validated questionnaires. We used logistic regression to explore independent predictors of meal‐related discomfort or pain ≥ 50% of the time in IBS.

**Key Results:**

Of 752 respondents with Rome IV IBS, 561 (74.6%) reported meal‐related abdominal discomfort or pain ≥ 50% of the time. 89.3% of individuals with meal‐related discomfort or pain ≥ 50% of the time were female vs. 80.6% of those without (*p* = 0.002). Those with meal‐related discomfort or pain ≥ 50% of the time were younger (43.7 years vs. 50.1 years, *p* < 0.001), had a higher prevalence of symptoms meeting criteria for functional dyspepsia (FD), especially postprandial distress syndrome (49.1% vs. 30.2%, *p* < 0.001), and reported higher gastrointestinal symptom‐specific anxiety scores, lower IBS‐related quality of life scores, and higher levels of activity impairment (*p* < 0.001 for all analyses). After logistic regression analysis, females, those meeting criteria for FD, younger individuals, and those reporting higher gastrointestinal symptom‐specific anxiety scores were more likely to report meal‐related discomfort or pain ≥ 50% of the time.

**Conclusions:**

Meal‐related abdominal discomfort or pain ≥ 50% of the time was associated with female sex, younger age, and comorbid FD. Better characterization and recognition of patients affected by meal‐related discomfort or pain may allow more personalized dietary and psychological interventions.

## Graphical abstract

Seventy‐five percent of patients with Rome IV IBS experienced meal‐related abdominal discomfort or pain ≥ 50% of the time. Females, younger individuals, and those with comorbid functional dyspepsia and higher gastrointestinal symptom‐specific anxiety scores were more likely to report frequent meal‐related abdominal discomfort or pain. Recognizing these patients may improve personalized management.

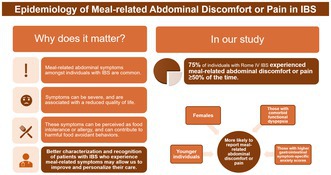

**Summary**

- Meal‐related abdominal discomfort or pain in IBS is common and not fully understood. We performed a cross‐sectional study among individuals with Rome IV‐defined IBS to examine their characteristics in relation to whether they experienced meal‐related abdominal discomfort or pain ≥ 50% of the time.
- 75% of individuals experienced meal‐related abdominal discomfort or pain ≥ 50% of the time. These individuals were more likely to be female, to be younger, to meet criteria for functional dyspepsia, and to report higher gastrointestinal symptom‐specific anxiety scores, lower IBS‐related quality of life scores, and higher levels of activity impairment from their IBS.
- Better characterization of patients with IBS who experience frequent meal‐related discomfort or pain may allow more personalized dietary and psychological interventions to help manage their symptoms.

**Abbreviations**
FODMAP: fermentable oligosaccharides, disaccharides, monosaccharides, and polyols
HADS: hospital anxiety and depression scale
IBS: irritable bowel syndrome
IBS‐C: irritable bowel syndrome with constipation
IBS‐D: irritable bowel syndrome with diarrhea
IBS‐M: irritable bowel syndrome with mixed bowel habits
IBS‐QOL: irritable bowel syndrome quality of life
IBS‐SSS: irritable bowel syndrome severity scoring system
IQR: interquartile range
PHQ‐12: patient health questionnaire‐12
VSI: visceral sensitivity index
WPAI: IBS: work productivity and activity impairment questionnaire: irritable bowel syndrome

## 1 Introduction

Irritable bowel syndrome (IBS) is a common disorder of gut‐brain interaction, characterized by recurrent abdominal pain and an altered bowel habit, affecting approximately 5% of the global population [1]. The pathophysiology is multifactorial, including interaction of mucosal and immune functions of the gut, visceral hypersensitivity, the gut microbiome, and central nervous system processing, although the interplay of these mechanisms is incompletely understood [2]. The psychosocial burden of IBS is substantial. 90% of patients report impairment of one or more areas of their lives across home management, social or leisure activities, and personal relationships. 80% report work impairment, losing an average of 2 h work per week due to their IBS [3], and there are well‐established associations between increasing IBS symptom severity and higher levels of psychological comorbidity [4].

Patients with IBS are also substantially affected in their dietary and meal experiences. 80% of patients report food intolerances [5, 6], often manifest as a precipitation or worsening of their symptoms after meals. Although the pathophysiological mechanisms underlying meal‐related symptoms are not fully understood, several food categories are implicated as triggers of gastrointestinal symptoms. These are primarily foods with high content of incompletely absorbed carbohydrates, or fermentable oligosaccharides, disaccharides, monosaccharides, and polyols (FODMAP), foods with high content of fats, biogenic amines and preservatives, as well as spicy foods, dairy, or wheat‐containing foods [5, 6, 7].

Patients with IBS frequently perceive their meal‐related symptoms must represent a specific food intolerance or allergy but struggle to identify a specific food trigger. Skin prick allergy testing and serological antibody testing have been unable to positively correlate foods perceived to be allergens with foods reported to be symptom triggers in patients with IBS [8, 9]. However, a recent study utilizing immunoglobulin G testing for 18 foods previously shown to elicit a response only in those with IBS rather than healthy controls found greater symptom relief with an antibody‐guided exclusion diet compared with a sham exclusion diet [10]. Higher reporting of food intolerance is associated with more severe IBS symptoms, higher levels of psychological comorbidity, and lower IBS‐related quality of life across multiple domains, including sleep, energy, diet, social functioning, and physical status [5]. It stands to reason that patients with more severe symptoms may be more likely to perceive intolerance to a greater number of foods, report greater disruptions to their life, and seek to restrict or exclude these foods as a means of managing their symptoms. This increases the potential risks of nutritional compromise and food avoidant behaviors, which more commonly affect those with more severe IBS and a higher burden of perceived food intolerance [11].

Current IBS management algorithms include exclusion diets, such as the low FODMAP diet. However, in some patients, this further compounds the perception that exclusion of dietary triggers should improve their symptoms. When this is not the case, it can lead to frustration and loss of confidence in care. Some patients seek food allergy testing privately, only to be further disappointed by the failure of such testing to identify consistent food triggers [9]. The specific subgroup of patients with IBS who experience frequent meal‐related symptoms remains incompletely characterized. Here, we aimed to compare the characteristics of adults with Rome IV‐defined IBS, with and without meal‐related abdominal discomfort or pain ≥ 50% of the time.

## 2 Materials and Methods

### 2.1 Participants and Setting

This cross‐sectional study recruited individuals registered with ContactME‐IBS, a UK national registry of over 4200 members with IBS who are interested in research, run by County Durham and Darlington NHS Foundation Trust [12]. ContactME‐IBS recruits individuals in the UK through advertisements in primary care, hospital clinics, pharmacies, or on social media. Those interested enroll by completing a short online questionnaire about their bowel symptoms and providing their contact details. Of the registrants, 2268 (53%) had seen a primary care physician with IBS, and another 1455 (34%) had seen a gastroenterologist. We have previously reported data from this cohort [3, 13, 14, 15, 16, 17]. All participants were contacted via electronic mailshot in July 2021, with nonresponders receiving a reminder email in August 2021. There were no exclusion criteria apart from the inability to understand written English. Responses were stored in an online database. Those completing the questionnaire were given a chance to win one of three gift cards worth £200, £100, or £50. The study was approved by the University of Leeds research ethics committee in March 2021 (MREC 20‐051).

### 2.2 Data Collection and Synthesis

#### 2.2.1 Demographic and Symptom Data

We collected demographic data, including age, sex, lifestyle factors such as tobacco and alcohol consumption, ethnicity, marital status, educational level, and annual income. We defined the presence of IBS using the Rome IV questionnaire [18], assigning presence of Rome IV‐defined IBS according to the scoring algorithm proposed for its use [19]. We categorized IBS subtypes using the proportion of time stools were abnormal according to the Bristol stool form scale. All participants also identified their most troublesome symptom from a list of five possibilities: abdominal pain; constipation; diarrhea; abdominal bloating or distension; or urgency. We asked participants about the length of time since their IBS diagnosis and whether their IBS started after an acute enteric infection. To identify those with overlapping functional dyspepsia (FD), we asked about the presence and frequency of early satiety, postprandial fullness, or epigastric pain or burning in the past 3 months, with onset of symptoms at least 6 months prior, using the Rome IV criteria for FD.

#### 2.2.2 IBS Symptom Severity

We assessed IBS symptom severity via the IBS severity scoring system (IBS‐SSS) [20], which measures presence, severity, and frequency of abdominal pain, presence and severity of abdominal distension, satisfaction with bowel habit, and degree to which IBS symptoms are affecting or interfering with the individual's life. The IBS‐SSS carries a maximum score of 500 points, with < 75 indicating remission of symptoms; 75–174 mild symptoms; 175–299 moderate symptoms; and 300–500 severe symptoms.

#### 2.2.3 Mood, Somatic Symptoms, and Gastrointestinal Symptom‐Specific Anxiety

We used the hospital anxiety and depression scale (HADS) to collect symptoms of anxiety and depression. The total HADS score ranges from 0 to 21 for either anxiety or depression [21]. We collected somatic symptom data using the patient health questionnaire‐12 (PHQ‐12) [22], derived from the validated PHQ‐15 [23]. The total PHQ‐12 score ranges from 0 to 24. We used the visceral sensitivity index (VSI) [24], which measures gastrointestinal symptom‐specific anxiety. Replies to each of the 15 items are provided on a 6‐point scale from “strongly disagree” (score 0) to “strongly agree” (score 5).

#### 2.2.4 IBS‐Specific and Generic Health‐Related Quality of Life

We used the irritable bowel syndrome quality of life (IBS‐QOL), a validated IBS‐specific questionnaire, to measure health‐related quality of life in individuals with IBS [25, 26]. The IBS‐QOL consists of 34 items, each ranked on a 5‐point Likert scale ranging from 0 to 4, with a total possible score of 0–136 and lower scores indicating better quality of life. We also used the EuroQOL [27], a generic health‐related quality of life questionnaire, used widely throughout healthcare. Specifically, we used the EQ‐5D‐5L instrument [28], which consists of five items covering different aspects of health: mobility; self‐care; ability to carry out usual activities; pain/discomfort; and anxiety/depression.

#### 2.2.5 IBS‐Related Resource Use

We collected data on healthcare usage related to a person's IBS over the 12 months prior to recruitment to the study. We applied costs for GP appointments from Unit Costs of Health and Social Care 2020 [29], and appointments, investigations, and unplanned inpatient days in secondary care using NHS 2019/20 National Cost Collection Data [30]. We applied the lowest price for a 1‐month supply of each IBS‐related medication using the online version of the British National Formulary (BNF) [31]. Further detail regarding this methodology is provided in our previous work [15].

#### 2.2.6 Impact of IBS on Productivity and Ability to Work

We used the work productivity and activity impairment questionnaire for irritable bowel syndrome (WPAI:IBS) [32], which is a validated questionnaire to assess the level of work productivity loss in people with IBS who are employed, as well as activity impairment in their activities of daily living. We also used the work and social adjustment scale (WSAS) [33], which has been used by others to measure the effect of IBS on individuals' ability to work, manage at home, engage in social and private leisure activities, and maintain close relationships [34, 35, 36, 37]. The five domains are scored on a 9‐point scale from “not at all” (score 0) to “very severely” (score 8).

### 2.3 Statistical Analysis

All participants who met Rome IV criteria for IBS were included in the analysis. We dichotomized participants according to the frequency of meal‐related discomfort or pain according to whether or not their discomfort or pain started or worsened after a meal ≥ 50% of the time. We compared the characteristics of participants according to IBS subtypes and most troublesome symptom. Categorical variables such as sex, IBS subtype, IBS symptom severity, and presence or absence of Rome IV FD were compared between individuals with and without meal‐related abdominal discomfort or pain ≥ 50% of the time using a *χ* ^2^ test. Data such as age, costs of IBS‐related resource use, and scores for mood and somatic symptoms, gastrointestinal symptom‐specific anxiety, quality of life, absenteeism, presenteeism, overall work impairment, or activity impairment were compared between groups using an independent samples *t*‐test or Mann–Whitney *U* test. Statistical significance was defined by a *p* value < 0.01. We performed a sensitivity analysis, dichotomizing presence of meal‐related abdominal pain or discomfort according to whether or not discomfort or pain started or worsened after a meal ≥ 70% of the time. We used logistic regression to explore predictors of meal‐related abdominal discomfort or pain ≥ 50% of the time, with the results reported using odds ratios (OR) with 95% confidence intervals (CI). All analyses were performed using SPSS for Windows (version 30.0 SPSS, Chicago, IL).

## 3 Results

**FIGURE 1:**
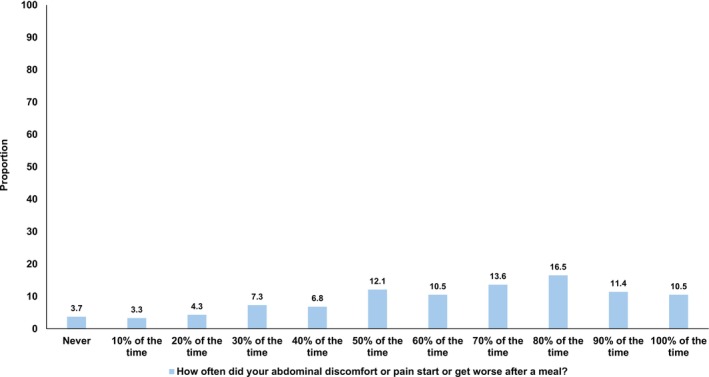
Frequency of meal‐related abdominal discomfort or pain in the last 3 months among 752 individuals with Rome IV IBS.

Of 4280 registrants in the ContactME‐IBS database, 1278 (29.9%) completed the questionnaire (mean age 47.2 years (range 18–89 years), 85% female). Of these, 752 (58.5%) met Rome IV criteria for IBS (mean age 45.3 years (range 18–81 years), 655 (87.1%) female and 729 (96.9%) white) and were included in the study. Of the 752 participants, 561 (74.6%) were affected by meal‐related abdominal discomfort or pain ≥ 50% of the time in the preceding 3 months (Figure 1). Of those with meal‐related abdominal discomfort or pain ≥ 50% of the time, 89.3% were female, compared with 80.6% of those without (*p =* 0.002). Those with meal‐related abdominal discomfort or pain ≥ 50% of the time were younger (43.7 years vs. 50.1 years, *p* < 0.001). There were no other statistically significant differences in demographic factors between those with and without meal‐related abdominal discomfort or pain ≥ 50% of the time, including marital status, smoking status, alcohol use, higher educational attainment, or having an annual income of £30,000 or more (Table 1).

**TABLE 1:** Characteristics of individuals with Rome IV IBS according to presence or absence of meal‐related abdominal discomfort or pain ≥ 50% of the time.

	Presence of meal‐related abdominal discomfort or pain ≥ 50% of the time (*n* = 561)	Absence of meal‐related abdominal discomfort or pain < 50% of the time (*n* = 191)	*p* *
Female (%)	501 (89.3)	154 (80.6)	0.002
Mean age (SD)	43.7 (14.3)	50.1 (15.2)	< 0.001
White ethnicity (%)	541 (96.4)	188 (98.4)	0.17
Married (%)	350 (62.4)	137 (71.7)	0.20
Smoker (%)	64 (11.4)	18 (9.4)	0.45
Alcohol user (%)	329 (58.6)	110 (57.6)	0.80
University or postgraduate level of education (%)	234 (41.7)	80 (41.9)	0.97
Annual income of £30,000 or more (%)	144 (28.3)	53 (30.8)	0.54
IBS subtype (%)			
IBS‐C	97 (17.5)	39 (20.6)	0.015
IBS‐D	245 (44.2)	61 (32.3)	
IBS‐M	212 (38.3)	89 (47.1)	
Most troublesome symptom (%)			
Abdominal pain	121 (21.6)	48 (25.1)	0.008
Constipation	30 (5.3)	23 (12.0)	
Diarrhea	90 (16.0)	27 (14.1)	
Bloating or distension	175 (31.2)	43 (22.5)	
Urgency	145 (25.8)	50 (26.2)	
IBS‐SSS severity (%)			
Mild	57 (10.2)	29 (15.4)	0.018
Moderate	216 (38.8)	84 (44.7)	
Severe	284 (51.0)	75 (39.9)	
IBS after acute infection (%)	64 (11.4)	27 (14.1)	0.32
IBS diagnosis for 5 years or more (%)	440 (78.4)	159 (83.2)	0.15
Seen a primary care physician regarding IBS in the last 12 months (%)	217 (38.7)	77 (40.3)	0.69
Seen a gastroenterologist regarding IBS in the last 12 months (%)	106 (18.9)	41 (21.5)	0.44
Number of IBS drugs in the last 12 months (%)			
0	71 (12.7)	25 (13.1)	0.36
1	131 (23.4)	58 (30.4)	
2	148 (26.4)	48 (25.1)	
3	97 (17.3)	32 (16.8)	
4	60 (10.7)	16 (8.4)	
≥ 5	54 (9.6)	12 (6.3)	
Functional dyspepsia (%)			
Epigastric pain syndrome present	195 (34.8)	39 (20.4)	< 0.001
Postprandial distress syndrome present	274 (49.1)	57 (30.2)	< 0.001
Mean VSI score (SD)	53.7 (15.5)	45.6 (17.8)	< 0.001
Mean HADS‐anxiety score (SD)	10.9 (4.7)	10.2 (5.1)	0.12
Mean HADS‐depression score (SD)	7.6 (4.4)	7.5 (4.8)	0.95
Mean PHQ‐12 score (SD)	10.6 (4.2)	9.7 (4.6)	0.014
Mean IBS‐QOL (SD)	46.3 (21.8)	54.4 (22.8)	< 0.001
Mean EQ‐5D (SD)	0.56 (0.28)	0.59 (0.29)	0.20
WSAS (%)			
IBS affects home management	170 (30.3)	50 (26.2)	0.28
IBS affects social leisure activities	327 (58.3)	96 (50.3)	0.05
IBS affects private leisure activities	162 (28.9)	45 (23.6)	0.16
IBS affects close relationships	160 (28.5)	43 (22.5)	0.11
WPAI:IBS, median (IQR)			
Absenteeism	0 (0–2.67)	0 (0–4.7)	0.92
Presenteeism	40 (20–60)	30 (10–60)	0.08
Overall work impairment	33.3 (10–60)	27.4 (6–51.9)	0.04
Activity impairment	50 (20–70)	30 (20–60)	< 0.001
Mean costs of IBS in £UK (SD)			
Appointments	226.90 (600.49)	217.40 (495.43)	0.84
Investigations	153.29 (357.99)	170.59 (335.71)	0.56
IBS‐related medications	74.87 (94.84)	66.00 (100.39)	0.27
Unplanned attendances	116.01 (473.59)	60.25 (260.05)	0.13
Total direct healthcare costs	571.08 (1082.62)	514.24 (828.88)	0.45

* *p* value for Pearson *χ* ^2^ for comparison of categorical data. Independent samples *t*‐test for continuous data, and Mann–Whitney *U* test for all four dimensions of WPAI:IBS.

In total, 136 (18.1%) participants had IBS‐C, 306 (40.7%) IBS‐D, and 301 (40.0%) IBS‐M. Those with IBS‐D had the highest prevalence of meal‐related abdominal discomfort or pain ≥ 50% of the time (44.2%), followed by those with IBS‐M (38.3%), then those with IBS‐C (17.5%). Most troublesome symptom reported differed significantly between those with meal‐related abdominal discomfort or pain and those without (*p* = 0.008); visual inspection suggested this related to a higher proportion of those with meal‐related abdominal discomfort or pain ≥ 50% of the time reporting bloating or distension as their most troublesome symptom (31.2% vs. 22.5%). There were no statistically significant differences in the proportion reporting onset of IBS symptoms following an acute enteric infection, a duration of IBS of 5 years or more, or review by a doctor in either primary or secondary care in the preceding 12 months between those with and without meal‐related abdominal discomfort or pain ≥ 50% of the time. The mean number of medications used for IBS in the prior 12 months was also not significantly different between the two groups.

Those with meal‐related abdominal discomfort or pain ≥ 50% of the time had a higher prevalence of Rome IV defined FD, especially postprandial distress syndrome (49.1% vs. 30.2%, *p* < 0.001). There were no significant differences in HADS‐anxiety or HADS‐depression scores or PHQ‐12 scores. Gastrointestinal symptom‐specific anxiety scores were significantly higher among those with meal‐related abdominal discomfort or pain ≥ 50% of the time (*p* < 0.001). Those with meal‐related abdominal discomfort or pain ≥ 50% of the time reported significantly lower IBS‐QOL scores, and significantly higher levels of activity impairment on the WPAI:IBS (*p* < 0.001 for both). There were no statistically significant differences in WSAS scores according to presence or absence of meal‐related abdominal discomfort or pain ≥ 50% of the time.

In sensitivity analysis, dichotomizing presence of meal‐related abdominal pain or discomfort according to whether or not discomfort or pain started or worsened after a meal ≥ 70% of the time, the findings were similar. A significantly higher proportion of individuals with meal‐related abdominal pain or discomfort were female (90.0% vs. 83.9%), met criteria for FD (postprandial distress syndrome 54.0% vs. 33.8%, epigastric pain syndrome 37.3% vs. 24.4%), and those with meal‐related abdominal pain or discomfort were significantly younger (mean 43.2 years vs. 47.6 years), had significantly higher gastrointestinal symptom‐specific anxiety scores (mean 55.2 vs. 47.8), significantly lower IBS‐related quality of life scores (mean 43.8 vs. 53.2), and significantly higher WPAI:IBS activity impairment scores (median 50 vs. 40).

We performed logistic regression analysis with all variables that were significantly associated with meal‐related abdominal discomfort or pain after univariate analysis entered into the model. Those reporting meal‐related abdominal discomfort or pain ≥ 50% of the time were significantly more likely to be female (OR 1.90; 95% CI 1.16–3.12) and meet criteria for Rome IV FD, including both postprandial distress syndrome (OR 1.60; 95% CI 1.07–2.39) and epigastric pain syndrome (OR 1.74; 95% CI 1.12–2.70). Older individuals (OR per year 0.98; 95% CI 0.97–0.99) were less likely to report meal‐related abdominal discomfort or pain ≥ 50% of the time and those with higher gastrointestinal symptom‐specific anxiety scores (OR per unit 1.03; 95% CI 1.01–1.04) more likely. Overall, the logistic regression model explained 15.6% of the variance of the data.

## 4 Discussion

This cross‐sectional survey examined the prevalence of meal‐related abdominal discomfort or pain ≥ 50% of the time among individuals with Rome IV IBS. Overall, 75% of individuals experienced meal‐related discomfort or pain affecting them 50% of the time or more. After univariate analysis, individuals with Rome IV IBS who reported meal‐related abdominal discomfort or pain ≥ 50% of the time were more likely to be female, to be younger, to meet criteria for FD, to have higher gastrointestinal symptom‐specific anxiety scores, to have worse IBS‐specific quality of life scores, and to report higher levels of activity impairment from their IBS. After logistic regression, female sex, meeting Rome IV criteria for FD, younger age, and reporting higher gastrointestinal symptom‐specific anxiety scores were independently associated with reporting meal‐related discomfort or pain ≥ 50% of the time.

There are some limitations of the study. We were unable to objectively exclude the presence of organic conditions that IBS may mimic, such as coeliac disease or inflammatory bowel disease. However, our cohort was recruited from a community setting, likely representing many patients with IBS and including those who had no recent contact with a primary or secondary care physician regarding their symptoms. Given that 80% of the study participants had had a diagnosis of IBS for 5 years or more, and that screening for these conditions is recommended in the IBS management guidelines, it would seem reasonable to assume that IBS is the correct diagnosis for these individuals. The study population was 97% white, limiting generalizability of the findings to other ethnicities. This could be particularly relevant as dietary and mealtime habits vary widely across ethno‐racial groups and cultural settings and may influence the perceived experience of meal‐related abdominal discomfort or pain. Although IBS is more common among females, the proportion of female individuals in our study is higher than might be expected. This might indicate that female individuals, together with those of white ethnicity, are more likely to register with the ContactME‐IBS database. However, the reasons for this are beyond the scope of the current study to determine. As this was a cross‐sectional survey, the direction of the effects we report cannot be ascertained. We recruited 752 participants with IBS, representing a 30% response rate to the invitation. This could be considered a low response rate. Although individuals who register with ContactMe‐IBS have expressed an interest in participating in research, it is not a requirement of their registration to do so. We acknowledge the risk of selection bias, as it is possible registrants with more severe IBS symptoms may have been more likely to participate. However, other studies have also reported a high prevalence of food and meal‐related gastrointestinal symptoms ranging from 50% in the general population [38], to 70%–80% in some cohorts of patients with IBS [6, 9], a similar prevalence to our study.

Female sex has been consistently observed to be associated with higher rates of reporting of food‐related symptoms [5, 7, 38], and our study further supports this. We have also replicated an association between the presence of meal‐related abdominal discomfort or pain and a lower quality of life observed in patients with IBS by others [5]. This is unsurprising considering the high importance individuals attach to their meal experiences, and has also been recognized in the general population in the Rome Foundation Global Epidemiological Study of over 18,000 individuals [38]. Similar to our findings, this study also found the presence of FD to be a predictor for reporting frequent meal‐related symptoms, and that bloating or distension was the most troublesome symptom in more individuals with frequent meal‐related symptoms, although younger age was also a significant association. In contrast, studies of Swedish and Korean patients with IBS have reported loose stools to be the most troublesome associated symptom [6, 11].

The positive association between increasing burden of meal‐related abdominal discomfort or pain and more severe IBS symptoms has previously been observed in patients in secondary and tertiary care settings [5]. Although our results suggested a trend towards an association, this did not reach statistical significance. This could reflect the community setting of our study, among whom we might reasonably expect less severe IBS symptoms, in view of their lower likelihood of contact with gastroenterology services. As per previous studies we did not find any association between IBS subtype and meal‐related abdominal discomfort or pain [5, 39], although Melchior et al. have reported diarrhea to be the most frequently reported bowel habit among patients with IBS with the most severe food avoidance behaviors [11]. Regarding psychological comorbidity, previous studies have found significant associations between more meal‐related symptoms and a greater burden of psychological disorders, such as depression and anxiety [7, 11, 40].

It is not surprising that patients with IBS often seek dietary intervention to manage their condition. Our elicitation of an independent association between higher gastrointestinal symptom‐specific anxiety scores and the reporting of meal‐related discomfort or pain ≥ 50% of the time supports this intuition. Although the distribution of scores according to response to specific questions across the components of the VSI was not examined here, 4 of the 15 questions pertain to anxieties centered on eating, a common concern for many patients in the clinical setting who have often already tried their own food exclusion trials. The most widely evidenced food exclusion intervention is the low FODMAP diet, as recommended in current IBS management algorithms, but there are other promising new dietary therapies emerging [41]. A greater number of viable options for efficacious dietary intervention may further enhance personalized approaches to treatment, for which there is strong advocacy [42, 43], although whether dietary interventions lead to an improvement in meal‐related symptoms, specifically, is unknown.

It has been suggested that patients with IBS with frequent meal‐related symptoms could represent a previously undefined subgroup, often with more severe symptoms and a greater degree of psychological and psychosomatic comorbidity [42]. Acknowledgement of the particular patient subset affected by frequent meal‐related symptoms is anticipated in the updated Rome V IBS diagnostic criteria, which may drive improved recognition of these patients. There is also increasing concern over the risk of food avoidant and restrictive behaviors among patients with IBS, particularly those with the most severe and intractable symptoms [11]. Exclusion diets may not be the most appropriate strategy for these individuals, and early recognition of those most at‐risk will allow early intervention with a view to limiting food‐related anxiety and preventing progression towards the most severe forms of food restriction, such as avoidant/restrictive food intake disorder.

In summary, the high prevalence of meal‐related abdominal discomfort or pain among IBS patients has been demonstrated consistently in several studies and warrants consideration as a core intervention target. Our study reveals that these symptoms are particularly associated with female sex, younger age, and comorbid FD. Better characterization and recognition of patients affected by meal‐related abdominal discomfort or pain may allow more targeted interventions to address symptoms and dietary habits. This requires holistic understanding of their symptom experience, and a multidisciplinary approach involving physicians, dietitians, and clinical psychologists, to provide more personalized management strategies.

## Author Contributions

M.S.C., V.C.G., C.E.N., C.J.B., and A.C.F. conceived and drafted the study. V.C.G. and C.E.N. collected all data. M.S.C. and V.C.G. analyzed and interpreted the data. M.S.C., C.J.B., and A.C.F. drafted the manuscript. All authors have approved the final draft of the manuscript.

## Conflicts of Interest

The authors declare no conflicts of interest.

## Data Availability

The data that support the findings of this study are available from the corresponding author upon reasonable request.
